# Public Knowledge, Attitudes, and Preventive Practices Regarding Mpox in Bangladesh: Insights From a Community‐Based Cross‐Sectional Survey

**DOI:** 10.1002/hsr2.73401

**Published:** 2026-10-16

**Authors:** Hemayet Hossain, Md. Hasan Ali, Md. Abu Boccor Siddique, Sumaya Shargin Khan, Milon Mia, Suchona Akter, Afroza Sultana Nitu, Mohammad Nahian Rahman, Mostafizor Rahman, Md. Abdur Nur Sakib, Md Mosharof Hosen, Jakia Sultana koly, Tanvir Ahmad, Sadia Islam Ria, Tanvir Ahmed, Khadiza Akter Brishty, Md. Nafij Mashrur, Shihab Ahmed, Md. Shahidur Rahman Chowdhury, Md. Mahfujur Rahman

**Affiliations:** ^1^ Department of Anatomy & Histology Sylhet Agricultural University Sylhet Bangladesh; ^2^ Faculty of Veterinary, Animal and Biomedical Sciences Sylhet Agricultural University Sylhet Bangladesh; ^3^ Faculty of Animal Science and Veterinary Medicine Patuakhali Science and Technology University Dumki Bangladesh; ^4^ Department of Medicine Sylhet Agricultural University Sylhet Bangladesh; ^5^ Faculty of Veterinary Medicine Chattogram Veterinary and Animal Sciences University Chattogram Bangladesh; ^6^ Department of Dairy and Poultry Science Hajee Mohammad Danesh Science and Technology University Dinajpur Bangladesh; ^7^ Department of Animal Nutrition Habiganj Agricultural University Habiganj Bangladesh; ^8^ Department of Chemistry Eden Mohila College, University of Dhaka Dhaka Bangladesh; ^9^ Department of Zoology (GSSC) University of Dhaka Dhaka Bangladesh; ^10^ Department of Biochemistry and Molecular Biology Gono Bishwabidyalay Ashulia Dhaka Bangladesh; ^11^ AMR Reference Laboratory (Research) Bangladesh Livestock Research Institute Savar Dhaka Bangladesh

**Keywords:** community‐survey, health behavior, Mpox, preventive practices, public awareness

## Abstract

**Background and Aims:**

Mpox has recently re‐emerged as a significant global health concern, highlighting the importance of public awareness and preventive measures. This study aimed to assess the knowledge, attitudes, and practices (KAP) regarding Mpox and its epidemiology, prevention, and control measures among the general population of Bangladesh.

**Methods:**

A community‐based cross‐sectional survey was conducted from June 2024 to September 2024 among 1002 participants across 16 districts, using a multi‐stage random sampling approach following STROBE guidelines. Data were collected through a structured questionnaire covering sociodemographics, knowledge, attitudes, and practices. Both univariate and multivariate logistic regression analyses were used to determine associations between sociodemographic factors and KAP outcomes.

**Results:**

Out of 1002 respondents, only 37.2% demonstrated good knowledge of Mpox, while 74.0% had positive attitudes, and 80.0% reported satisfactory preventive practices. Awareness of Mpox as a viral disease was limited (37.3%), and only 22.5% knew about the protective role of smallpox vaccination. Higher education was strongly associated with good knowledge (AOR = 11.8; 95% CI: 5.61–25.8), positive attitudes (AOR = 5.68; 95% CI: 2.63–12.5), and satisfactory practices (AOR = 6.49; 95% CI: 2.62–16.8). Non‐border residents were more likely to report positive attitudes (AOR = 3.07; *p* < 0.001) and satisfactory practices (AOR = 3.84; *p* < 0.001). Urban (AOR = 2.28; *p* = 0.003) and semi‐urban (AOR = 2.19; *p* = 0.014) participants showed more positive attitudes. Attitudes and practices showed the strongest correlation (*ρ* = 0.793, *p* < 0.001), indicating a strong association between attitudes and preventive practices.

**Conclusion:**

The findings suggest that gaps in awareness and training are associated with suboptimal practices, highlighting potential areas for targeted educational interventions. Strengthening community‐level awareness and preparedness may support public health readiness for potential Mpox outbreaks in Bangladesh.

## Summary

- This study provides the first large‐scale evidence of how people in Bangladesh understand and respond to Mpox, revealing that while most have positive attitudes and preventive habits, their basic knowledge remains limited.
- Higher educational attainment was strongly associated with awareness and preventive practices, highlighting the potential value of strengthening public health education to support community preparedness.
- The findings highlight the urgent need for awareness campaigns in border and low‐education areas to reduce the risk of Mpox introduction and improve readiness for future zoonotic disease outbreaks.

## 1 Introduction

In addition to the COVID‐19 pandemic, the world is currently facing a global outbreak of monkeypox (Mpox). In May 2022, over 3200 Mpox cases were reported across more than 50 countries, prompting the World Health Organization (WHO) to designate Mpox an “evolving danger of considerable health concern” in June 2022 [1]. The virus was first identified in 1958 in Denmark, when it was isolated from captive cynomolgus monkeys (*Macaca fascicularis*), which gave rise to the name monkeypox [2].

Mpox is a zoonotic viral disease caused by the monkeypox virus, a member of the genus Orthopoxvirus under the family Poxviridae [3, 4]. The first human case was reported in 1970 in the Democratic Republic of Congo [5]. Since then, numerous outbreaks have occurred in Central and West Africa, and the disease has now spread to other parts of the world [6]. The first outbreak outside Africa was documented in the United States in 2003, linked to imported exotic pets from Ghana [7]. The global expansion of Mpox has raised concerns about its potential public health impact [8]. Over the past two decades, human Mpox cases have significantly increased [9], making it one of the most important orthopoxviruses of public health concern [10]. The discontinuation of smallpox vaccination, which provided cross‐protection against Mpox, has likely contributed to this rising incidence [11].

Animal reservoirs include rodents such as rats and rabbits, and non‐human primates, with transmission occurring through direct contact with blood, body fluids, or lesions of infected animals, or indirectly via handling bushmeat or animal products [12]. Human‐to‐human transmission primarily occurs through direct contact, respiratory droplets, and contaminated materials. After viral entry, replication occurs at the inoculation site, followed by systemic spread [13]. The incubation period is 6–13 days, with an invasion stage lasting up to 5 days, characterized by fever, headache, myalgia, back pain, chills, fatigue, and lymphadenopathy [14]. Severe disease is more likely in children, pregnant women, and immunocompromised individuals [15]. Complications include secondary infections, respiratory distress, sepsis, encephalitis, corneal infections with potential vision loss, and gastrointestinal disturbances [3, 16].

Endemic Mpox outbreaks have been reported in several African countries, including the Democratic Republic of Congo, Republic of Congo, Central African Republic, Liberia, Nigeria, and Cameroon [9, 17]. More recently, cases have been reported outside Africa, including in the United States, the United Kingdom, Israel, and Singapore [15, 18, 19]. Although no confirmed case has been reported in Bangladesh to date, the government declared a state of emergency in 2022 in response to the global outbreak [20].

Public awareness and knowledge are critical for outbreak preparedness. Previous knowledge, attitudes, and practices (KAP) studies from Saudi Arabia, Indonesia, and Jordan revealed limited public understanding of Mpox [21, 22]. In Bangladesh, several risk factors for potential introduction exist, including porous borders and animal movement. Inadequate enforcement of the Animal Trespass Act allows cross‐border movement of animals from India into Bangladesh, raising the risk of transboundary zoonotic disease spread [23]. Historical data suggest that imported cases are often responsible for outbreaks outside Africa [24].

Environmental and demographic conditions also contribute to risk. Living in forested areas with animal reservoirs, particularly squirrels and monkeys, has been associated with infection risk in Africa [25]. Non‐invasive interactions, such as handling, cleaning cages, or preparing bushmeat, can facilitate zoonotic transmission [26]. Risk factors such as younger age, male gender, and lack of prior smallpox vaccination further increase susceptibility.

Given the rising global incidence, understanding the KAP of populations in high‐risk areas is essential [27].

To the best of our knowledge, this is the first study conducted in Bangladesh to assess the factors associated with the KAP, as well as behavioral factors associated with Mpox, among the study Population. Therefore, this study aims to evaluate the KAP related to Mpox among the Bangladeshi population, with a particular focus on border and host‐exposed areas. By identifying existing knowledge gaps, misconceptions, and preventive behaviors, the study seeks to inform public health preparedness and guide interventions to mitigate the risk of future Mpox outbreaks in Bangladesh.

## 2 Materials and Methods

### 2.1 Ethics Statement

This study was conducted in accordance with the ethical principles outlined in the Declaration of Helsinki, ensuring respect, confidentiality, and voluntary participation of all respondents. Participants gave written consent, indicating that they understood the study's goal and their rights. Confidentiality procedures were tightly enforced to preserve participants' privacy, and every attempt was made to reduce any potential risk or discomfort. This study also obtained ethical clearance from the “Institutional Ethical Committee” of Sylhet Agricultural University, Bangladesh, with approval number: #ARP2025030.

### 2.2 Study Design, Location, and Sampling Strategy

A community‐based cross‐sectional survey was carried out among 1002 participants in Bangladesh between June 2024 and September 2024 following STROBE guidelines (Supporting Information S1: Checklist S1). Bangladesh is administratively divided into eight divisions, which include central, coastal, and border regions. For this study, a multi‐stage random sampling strategy was employed. For operational classification, districts adjacent to the Bay of Bengal were categorized as coastal regions, districts sharing international boundaries with neighboring countries were categorized as border regions, and the remaining inland districts were classified as central regions.

In the first stage, two districts were selected from each division, ensuring that at least four districts were represented from each geographic category (central, coastal, and border areas). This process yielded a total of 16 districts. In the second stage, 3 upazilas (sub‐districts) were randomly chosen from each selected district, resulting in 48 upazilas overall (Figure 1).

**Figure 1:**
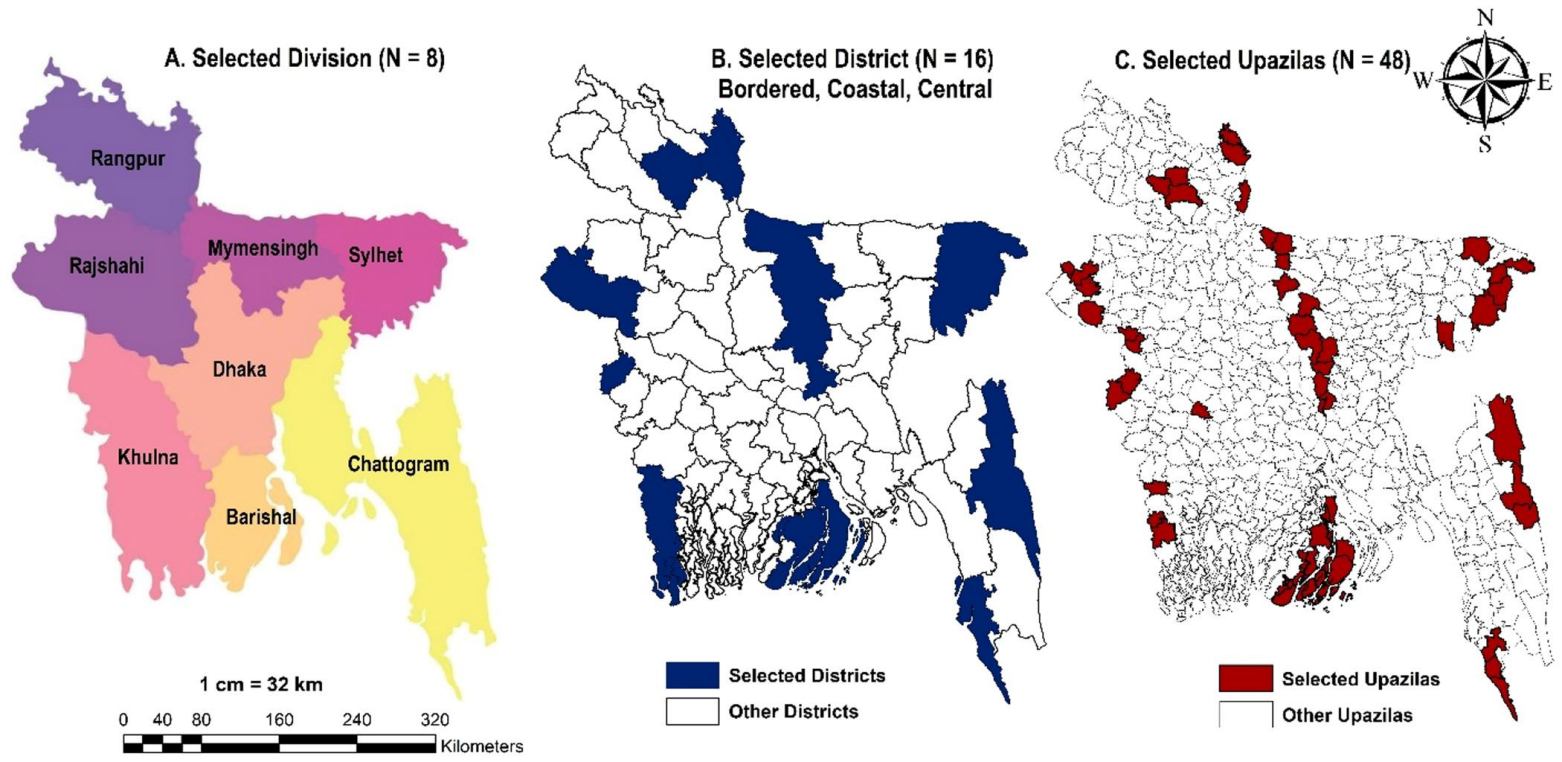
Geospatial distribution of the study areas included in the community‐based Mpox KAP survey conducted in Bangladesh from June to September 2024. (A) Selected division of the study (*N* = 8). (B) Selected districts (*N* = 16) based on coastal, bordered, and central monkey exposed area. (C) Selected upazilas (*N* = 48) among the districts. The map was generated using ArcMap 10.8 software.

Before participant recruitment, a household sampling frame was developed for each selected upazila through local household listings and field verification conducted by the research team. Systematic random sampling was subsequently employed to select households within each upazila. The sampling interval (*k*) was calculated by dividing the estimated total number of households in the selected area by the required sample size for that upazila. A random starting point was selected, and thereafter every *k* ^th^ household was approached sequentially for inclusion in the survey.

Eligible participants were adults aged 18 years or older who were permanent residents of the selected household, capable of understanding the survey questions, and willing to provide informed consent. Individuals who were severely ill or unable to complete the interview were excluded from participation. In households with more than one eligible participant, one respondent was selected using a simple random selection procedure to minimize potential selection bias.

Finally, a minimum of 20 respondents were interviewed from each upazila to achieve the target sample size.

The sample size was calculated using Cochran's formula for cross‐sectional studies with a 95% confidence level, a 5% margin of error, and an assumed population proportion of 50% [28]:

$$\text{Minimum required sample},\;n=\frac{Z^{2}\times P_{exp}\times \left(1-P_{exp}\right)}{d^{2}},$$

where, *n* = desired sample size; *Z* = 1.96 for 95% confidence interval (CI); *P*_exp_ = 0.5, expected positive outcome (50%); *d* = 0.05.

Based on this calculation, the minimum required sample size was 384. However, 1002 participants were ultimately surveyed to enhance precision and representativeness.

### 2.3 Questionnaire Preparation

The questionnaire was developed based on previously published literature [29, 30, 31, 32], with necessary modifications and inputs from an expert panel. It comprised four domains (Supporting Information S2: Questionnaire S1):

(A) Sociodemographic profile including age, gender, education level, occupation, income, household size, participant's settlement type (rural, semi‐rural, urban), location upazila (bordered, non‐bordered, coastal, central), previous health issues (related to skin rash, fever, muscle pain), and infected by any type of pox before (or similar signs of pox). (B) Knowledge: A total of 15 questions addressing etiology, transmission, epidemiology, and prevention/control measures of Mpox. (C) Attitude: A total of 14 questions assessing participants' perceptions and attitudes toward Mpox infection and prevention. (D) Practice: A total of 14 questions assessing preventive and control‐related practice. The questionnaire was prepared in both Bengali (local language) and English.

### 2.4 Data Collection and Survey

The survey was conducted through face‐to‐face interviews, with each participant interviewed for a minimum of 20 min. A pilot study involving 50 participants from the target population was carried out to assess the questionnaire's (Supporting Information S2: Questionnaire S1) clarity, acceptability, and overall comprehensibility, ensuring its suitability for the main survey. Following the pilot testing, minor revisions were implemented to refine the questionnaire based on participant feedback and observed inconsistencies. Data obtained from the pilot trial were excluded from the final analysis. Data collection was carried out by trained researchers' assistants under strict confidentiality protocols to ensure the privacy and protection of the participants' information. The survey was conducted in the local language by trained enumerators. During each interview, one enumerator asked the questions while another observed and recorded the responses. Data were documented digitally using the KoboToolbox platform.

### 2.5 Response to the Knowledge, Attitude, and Practice Assessment

Knowledge was evaluated by assigning 2 points for correct responses, 1 point for irregular responses, and 0 points for incorrect or uncertain answers. Attitude and practice‐based questions were measured using a five‐point Likert scale: Strongly Agree (5), Agree (4), Neutral (3), Disagree (2), and Strongly Disagree (1). The overall KAP scores were derived by aggregating participants' responses within each respective domain, resulting in score ranges of 0–30 for knowledge, 0–70 for attitude, and 0–70 for practice. Subsequently, each participant's score was converted into a percentage by dividing the obtained score by the maximum possible score for the respective domain and multiplying by 100. A 65% cut‐off point was applied to the percentage scores to categorize participants into higher and lower levels of knowledge, attitude, and practice [33]. Scores equal to or above the cut‐off point were categorized as good knowledge, positive attitude, and satisfactory practices, while scores below 65% indicated poor knowledge, negative attitudes, and unsatisfactory practices. The estimation of overall KAP levels was conducted based on previously established methods [33, 34].

### 2.6 Geo‐Spatial Mapping

The surveyed divisions, districts, and upazilas were visualized in Figure 1. To illustrate the study area, geospatial mapping was performed using ArcMap version 10.8 (ESRI, Redlands, CA, USA). Each selected upazila (sub‐district) was marked and highlighted to indicate the specific locations where data were collected.

### 2.7 The Reliability of the Questionnaire

We carried out a thorough assessment of the validity and internal consistency of the questionnaire in order to verify its accuracy and ensure the dependability of our data acquisition method. Cronbach's alpha values were used to assess the questionnaire's internal consistency. IBM SPSS software was used to calculate the Cronbach's alpha values. The alpha score of the knowledge sections was 0.956, the attitude sections were 0.941, and the practice sections were 0.959. A Cronbach's alpha number above 0.60 denotes an adequate level of reliability, whereas a value above 0.80 denotes an exceptional level [35].

### 2.8 Statistical Analysis

At the end of data collection, the complete questionnaire was manually reviewed to verify the accurate completion of variables and data quality, before being cleaned and scored using Microsoft Excel (Microsoft 365 version). All analyses were conducted at the individual level, with each participant's KAP scores calculated separately and used as the primary unit of analysis for statistical modeling. Although the study employed a multistage sampling design, the statistical analyses assumed independence of observations, and potential clustering effects were not explicitly adjusted for in the analysis.

All statistical analyses were conducted using R Programming (version 4.4.2). Reproducible descriptive tables, univariable and multivariable logistic regression tables, were generated using the gtsummary‐R package for reproducible research [36].

Descriptive statistics, including frequencies and percentages, were employed to summarize categorical variables. Univariable and multivariable binary logistic regression analyses were conducted to examine the association between sociodemographic factors (independent variables) and dichotomous outcome variables, including knowledge, attitude, and practice levels (dependent variables), using a significance level of *p* < 0.05. Univariable logistic regression analysis was employed to estimate crude odds ratios and corresponding 95% CIs for various sociodemographic variables. To achieve comprehensive adjustment, all sociodemographic factors were incorporated into the multivariable logistic regression model, irrespective of their significance in the univariable analysis. A multivariable logistic regression model was used to estimate adjusted odds ratios (AORs) along with their corresponding 95% CIs. Statistical significance was defined as a p‐value less than 0.05, and results were reported as AORs accompanied by 95% CIs. Furthermore, Spearman's rank correlation coefficient was utilized to assess the relationships among KAP.

## 3 Results

### 3.1 Sociodemographic Characteristics of Respondents

A total of 1002 participants were surveyed (Table 1). Of these, 54.9% were male, and 45.1% were female. The majority (42.2%) held a bachelor's degree or higher, while 20.8% had no formal education. The most common occupations were students (28.3%), day laborers (20.1%), and housewives (17.9%). Approximately one‐third of respondents reported no monthly income (33.2%). Most participants lived in rural areas (54.0%), with 29.1% from border regions, 17.4% from coastal areas, and 7.6% from central regions. The largest age group was 20 to 30 years (52.1%). More than two‐thirds (71.2%) reported having been infected with some type of pox (or similar symptoms) in the past, and 44.8% reported previous health issues related to skin rash, fever, or muscle pain.

**Table 1:** Sociodemographic characteristics of respondents (*N* = 1002).

	Frequency (%)	
Variables	*n* (%)	95% CI
Participant gender		
Male	550 (54.9%)	51.8–85.0
Female	452 (45.1%)	42.0–48.3
Age (years)		
< 20	47 (4.7%)	3.5–6.2
20–30	522 (52.1%)	48.9–55.2
31–40	211 (21.1%)	18.6–23.7
40+	222 (22.2%)	19.6–24.9
Education level		
No formal education	208 (20.8%)	18.3–23.4
Primary education	102 (10.2%)	8.4–12.2
Secondary/Higher secondary	269 (26.8%)	24.1–29.7
BSc Hons or higher degree	423 (42.2%)	39.1–45.3
Occupation		
Student	284 (28.3%)	25.6–31.2
Housewife	179 (17.9%)	15.5–20.4
Day laborer	201 (20.1%)	17.6–22.7
Business person	156 (15.6%)	13.4–17.9
Healthcare provider	112 (11.2%)	9.3–13.3
Government employee	70 (7.0%)	5.5–8.7
Income level/month (BDT TK)		
No income	333 (33.2%)	30.3–36.3
Below 10,000	239 (23.9%)	21.2–26.6
10,000–30,000	287 (28.6%)	25.8–31.6
30,000–50,000	116 (11.6%)	9.7–13.7
Over 50,000	27 (2.7%)	1.8–3.9
Household size (number of family members)		
Below 4	163 (16.3%)	14.0–18.7
4–5	589 (58.8%)	55.7–61.9
5+	250 (25.0%)	22.3–27.8
Participant's settlement type		
Rural	541 (54.0%)	50.9–57.1
Semi‐urban	190 (19.0%)	16.6–21.5
Urban	271 (27.0%)	24.3–29.9
Location upazila		
Bordered	292 (29.1%)	26.3–32.1
Non‐bordered	460 (45.9%)	42.8–49.1
Costal	174 (17.4%)	15.1–19.8
Central	76 (7.6%)	6.0–9.4
Previous health issues (related to skin rash, fever, muscle pain)		
Yes	449 (44.8%)	41.7–47.9
No	553 (55.2%)	52.0–58.3
Infected by any type of pox before (or similar signs of Pox)		
Yes	713 (71.2%)	68.2–73.9
No	289 (28.8%)	26.0–31.8

*Note: N*: No. of total participants; *n*: No. of participants from different sections.

### 3.2 Knowledge of Mpox

Overall, 37.2% of respondents demonstrated a good level of knowledge, while 62.8% had poor knowledge (Table 2). Regarding specific knowledge items (Supporting Information S3: Table S1), only 54.3% had ever heard about Mpox. Awareness of its viral etiology was low, with 37.3% identifying Mpox as a viral disease, while 55.5% reported not knowing. Less than half (41.7%) were aware that outbreaks had occurred globally, including in Bangladesh.

**Table 2:** Overall level of knowledge, attitude, and practice regarding Mpox (level of measures).

	Frequency (%)	95% CI
Knowledge level		
Good	373 (37.2%)	34.2–40.3
Poor	629 (62.8%)	59.7–65.7
Attitude level		
Positive	741 (74.0%)	71.1–76.7
Negative	261 (26.0%)	23.3–28.9
Practice level		
Satisfactory	802 (80.0%)	77.4–82.5
Unsatisfactory	200 (20.0%)	17.5–22.6

Knowledge about transmission and prevention was limited: 40.5% knew Mpox could be transmitted from animals to humans, 41.0% recognized person‐to‐person transmission through direct contact, and 41.9% were aware that isolation of infected persons could prevent spread.

Only 22.7% were familiar with public health guidelines for prevention. Awareness of vaccination was poor, with just 22.5% knowing that smallpox vaccination may provide some protection. Nonetheless, 58.6% stated they would visit a doctor if they suspected Mpox infection (Figure 2).

**Figure 2:**
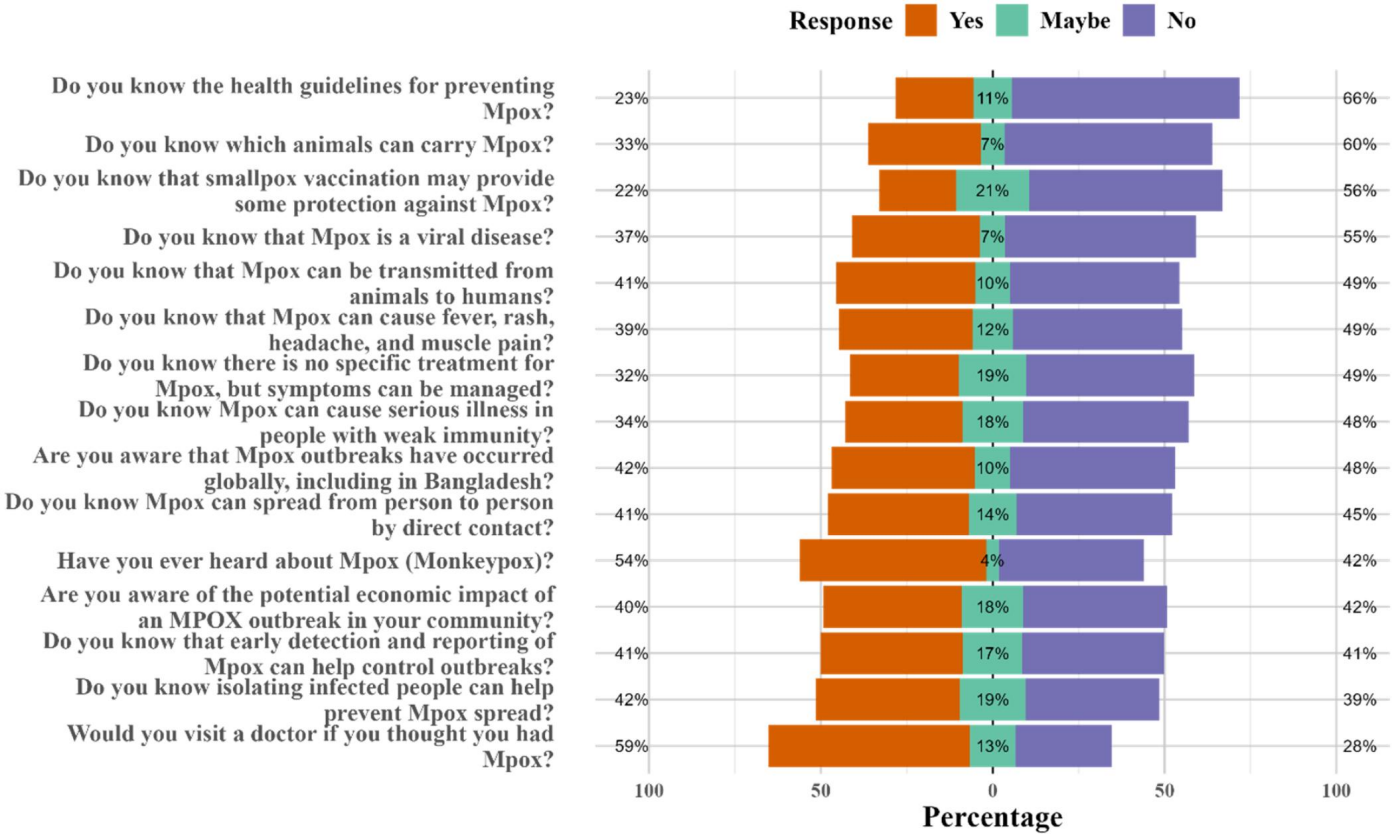
Knowledge‐related responses regarding Mpox among study participants in Bangladesh (*N* = 1002). The figure illustrates participants' awareness of Mpox etiology, transmission, prevention, vaccination, and healthcare‐seeking behavior. Values are presented as percentages of respondents.

### 3.3 Attitudes Towards Mpox

The majority of respondents (74.0%) held a positive attitude toward Mpox prevention and control, while 26.0% expressed negative attitudes (Table 2). Attitude‐specific responses (Supporting Information S4: Table S2) showed that 61.1% agreed Mpox poses a serious health risk, and 75.3% believed proper public health measures could control outbreaks. Nearly three‐quarters (77%) agreed that public awareness campaigns are important. However, perceptions of vaccination were mixed: only 36.3% agreed that smallpox vaccination provides protection, while 45.4% remained neutral. Concern about community spread was moderate, with 49.3% expressing concern. Regarding policy, 58.9% considered government efforts insufficient, while 54.5% agreed that people living near borders face higher risks. A large proportion also supported cross‐border (50.0%) and global collaboration (70.0%) in controlling Mpox (Figure 3).

**Figure 3:**
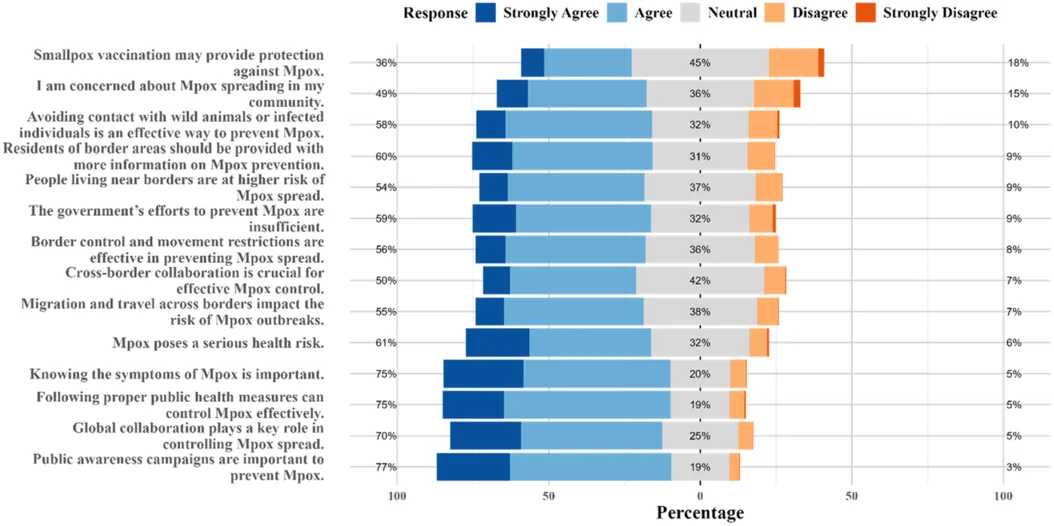
Distribution of attitude of participants regarding Mpox (*N* = 1002). The bar chart illustrates participants' responses to attitude‐related statements about Mpox. Agreement (agree and strongly agree) is represented on the left side of the axis, while disagreement (disagree and strongly disagree) is shown on the right.

### 3.4 Practices Towards Mpox Prevention

Most respondents (80.0%) reported satisfactory preventive practices, while 20.0% had unsatisfactory practices (Table 2). Practice‐specific findings (Supporting Information S5: Table S3) revealed that 78.4% agreed on the importance of handwashing after contact with animals or infected individuals, and 61.7% agreed on using personal protective equipment (Figure 4).

**Figure 4:**
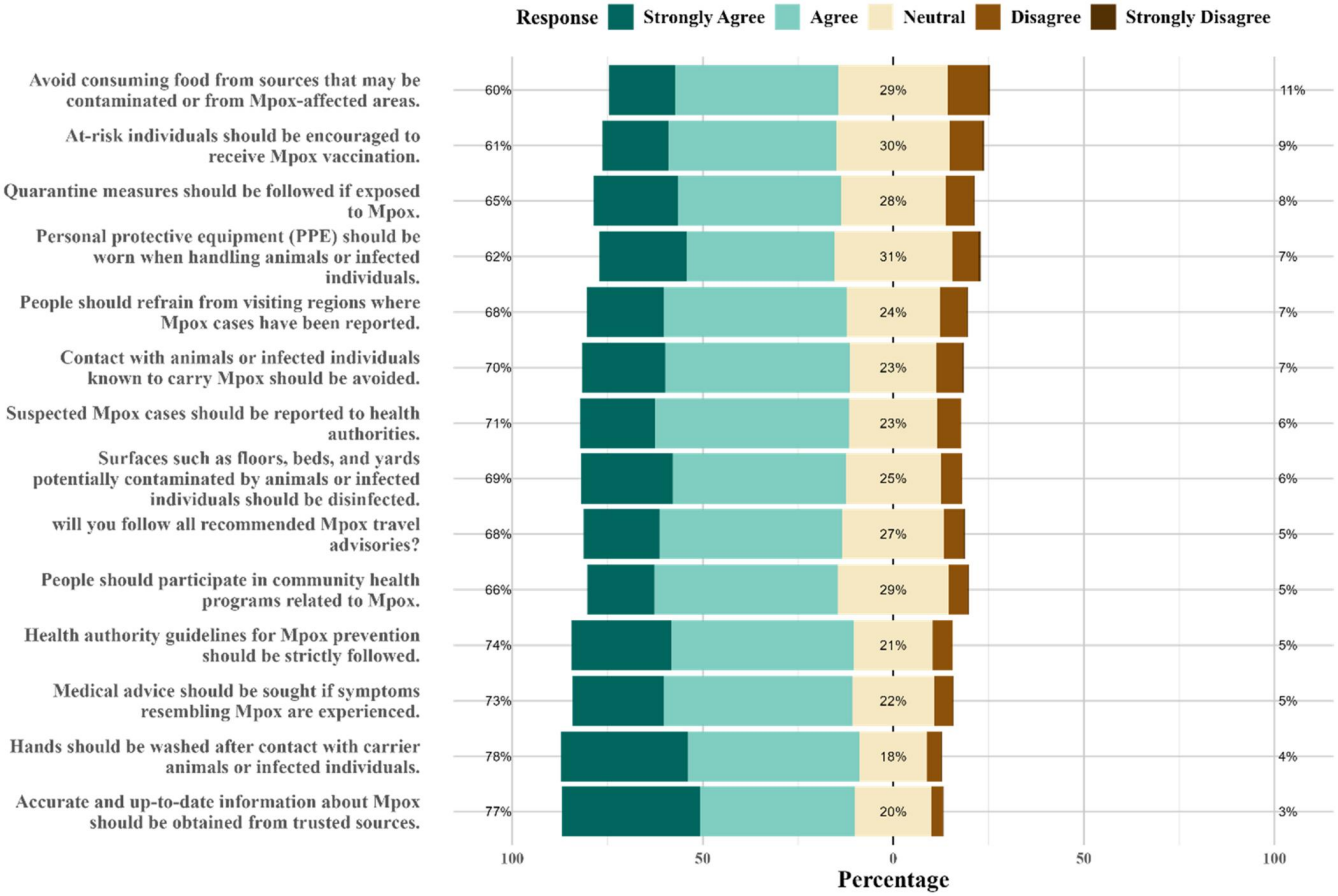
Distribution of practice of participants regarding Mpox (*N* = 1002). The bar chart illustrates participants' responses to practice‐related statements about Mpox. Agreement (agree and strongly agree) is represented on the left side of the axis, while disagreement (disagree and strongly disagree) is shown on the right.

Disinfection of contaminated surfaces was supported by 69.5%. Health‐seeking behavior was encouraging, with 73.5% agreeing that medical advice should be sought if symptoms occur. Furthermore, 70.3% supported avoiding contact with suspected cases, 74.1% endorsed following official guidelines, and 70.7% agreed suspected cases should be reported to health authorities. Travel precautions were also supported, with 68.3% agreeing to refrain from visiting outbreak regions. Importantly, 76.8% emphasized obtaining accurate information from trusted sources (Figure 4).

### 3.5 Factors Associated With KAP Levels

#### 3.5.1 Knowledge

Education was the strongest factor of Mpox‐related knowledge (Supporting Information S6: Table S4). Participants with a bachelor's degree or higher were over 11 times more likely to have good knowledge compared to those without formal education (AOR = 11.8; 95% CI: 5.61–25.8; *p* < 0.001), while secondary/higher secondary education also showed a positive association (AOR = 2.36; 95% CI: 1.23–4.69; *p* = 0.012). Larger households (≥ 5 members) were less likely to demonstrate good knowledge (AOR = 0.51; 95% CI: 0.34–0.77; *p* = 0.002). Coastal residents had higher odds of good knowledge than border residents (AOR = 2.24; 95% CI: 1.31–3.85; *p* = 0.003). Age ≥ 40 was linked with lower knowledge, though not significant after adjustment (AOR = 0.44; *p* = 0.086). Gender, income, and previous health issues were not significantly associated with knowledge.

#### 3.5.2 Attitudes

Education was also associated with attitudes (Supporting Information S7: Table S5), with respondents holding a bachelor's degree or higher nearly six times more likely to report positive attitudes (AOR = 5.68; 95% CI: 2.63–12.5; *p* < 0.001). Semi‐urban (AOR = 2.19; 95% CI: 1.19–4.20; *p* = 0.014) and urban residents (AOR = 2.28; 95% CI: 1.34–3.97; *p* = 0.003) were more likely to report positive attitudes than rural residents, and those from non‐border upazilas were more favorable than border residents (AOR = 3.07; 95% CI: 1.99–4.79; *p* < 0.001). Respondents with prior health issues also expressed more positive attitudes (AOR = 2.02; 95% CI: 1.36–3.02; *p* < 0.001).

#### 3.5.3 Practices

Satisfactory practices were most strongly associated with education (Supporting Information S8: Table S6). Respondents with a bachelor's degree or higher were over six times more likely to follow satisfactory practices (AOR = 6.49; 95% CI: 2.62–16.8; *p* < 0.001), with secondary/higher secondary education also significant (AOR = 1.95; 95% CI: 1.12–3.41; *p* = 0.019). Females had higher odds of satisfactory practices (AOR = 2.18; 95% CI: 1.17–4.16; *p* = 0.016). Day laborers were less likely to adopt satisfactory practices (AOR = 0.42; 95% CI: 0.20–0.84; *p* = 0.015).

Geographically, urban residents (AOR = 2.29; 95% CI: 1.20–4.65; *p* = 0.016) and non‐border upazila residents (AOR = 3.84; 95% CI: 2.30–6.51; *p* < 0.001) were more likely to report satisfactory practices, while coastal residents were less likely (AOR = 0.48; 95% CI: 0.28–0.81; *p* = 0.006). A history of health issues also increased the likelihood of satisfactory practices (AOR = 2.01; 95% CI: 1.28–3.19; *p* = 0.003). Previous pox infection history showed no significant effect.

A concise summary of the significant factors identified in the multivariable analyses is presented in Table 3. Across all KAP domains, higher educational attainment consistently showed the strongest association with favorable outcomes. Urban and non‐border residents generally demonstrated more positive attitudes and preventive practices, while larger household size and day laborer occupation were associated with poorer outcomes.

**Table 3:** Summary of key factors associated with Mpox‐related knowledge, attitudes, and practices among respondents in Bangladesh.

Variable	Knowledge		Attitude		Practice	
	AOR (95% CI)	*p* value	AOR (95% CI)	*p* value	AOR (95% CI)	*p* value
Participant gender						
Male					*Ref*.	
Female	—	—	—	—	2.18 (1.17, 4.16)	0.016
Education level						
No formal education	*Ref*.		*Ref*.		*Ref*.	
Primary education	—	—	—	—	2.42 (1.34, 4.46)	0.004
Secondary/Higher secondary	2.36 (1.23, 4.69)	0.012	—	—	1.95 (1.12, 3.41)	0.019
BSc Hons or higher degree	11.8 (5.61, 25.8)	< 0.001	5.68 (2.63, 12.50)	< 0.001	6.49 (2.62, 16.8)	< 0.001
Household size (number of family members)						
4–5	*Ref*.		—	—	—	—
Below 4	—	—	—	—	—	
5+	0.51 (0.34, 0.77)	0.002	—	—	—	—
Participant's settlement type						
Rural			*Ref*.		*Ref*.	
Semi‐urban	—	—	2.19 (1.19, 4.20)	0.01	2.17 (1.02, 5.09)	0.05
Urban	—	—	2.28 (1.34, 3.97)	0.003	2.29 (1.20, 4.65)	0.016
Location upazila						
Bordered	*Ref*.		*Ref*.		*Ref*.	
Non‐bordered	1.13 (0.75, 1.70)	0.6	3.07 (1.99, 4.79)	< 0.001	3.84 (2.30, 6.51)	< 0.001
Central	1.85 (0.98, 3.53)	0.05	—	—	—	—
Costal	2.24 (1.31, 3.85)	0.003	—	—	0.48 (0.28, 0.81)	0.006
Previous health issues (related to skin rash, fever, muscle pain)						
No			*Ref*.		*Ref*.	
Yes	—	—	2.02 (1.36, 3.02)	< 0.001	2.01 (1.28, 3.19)	0.003

*Note:* Only statistically significant factors (*p* < 0.05) from multivariable logistic regression models are presented.

### 3.6 Correlation Among Knowledge, Attitude, and Practice

A scatterplot correlation matrix with histograms was constructed to examine the relationships between respondents' knowledge, attitude, and practice regarding Mpox (Figure 5; Table 4). The diagonal plots show how each variable is distributed. The lower panels display scatterplots along with fitted trend lines. The upper panels display the Spearman's correlation coefficients and their significance levels.

**Figure 5:**
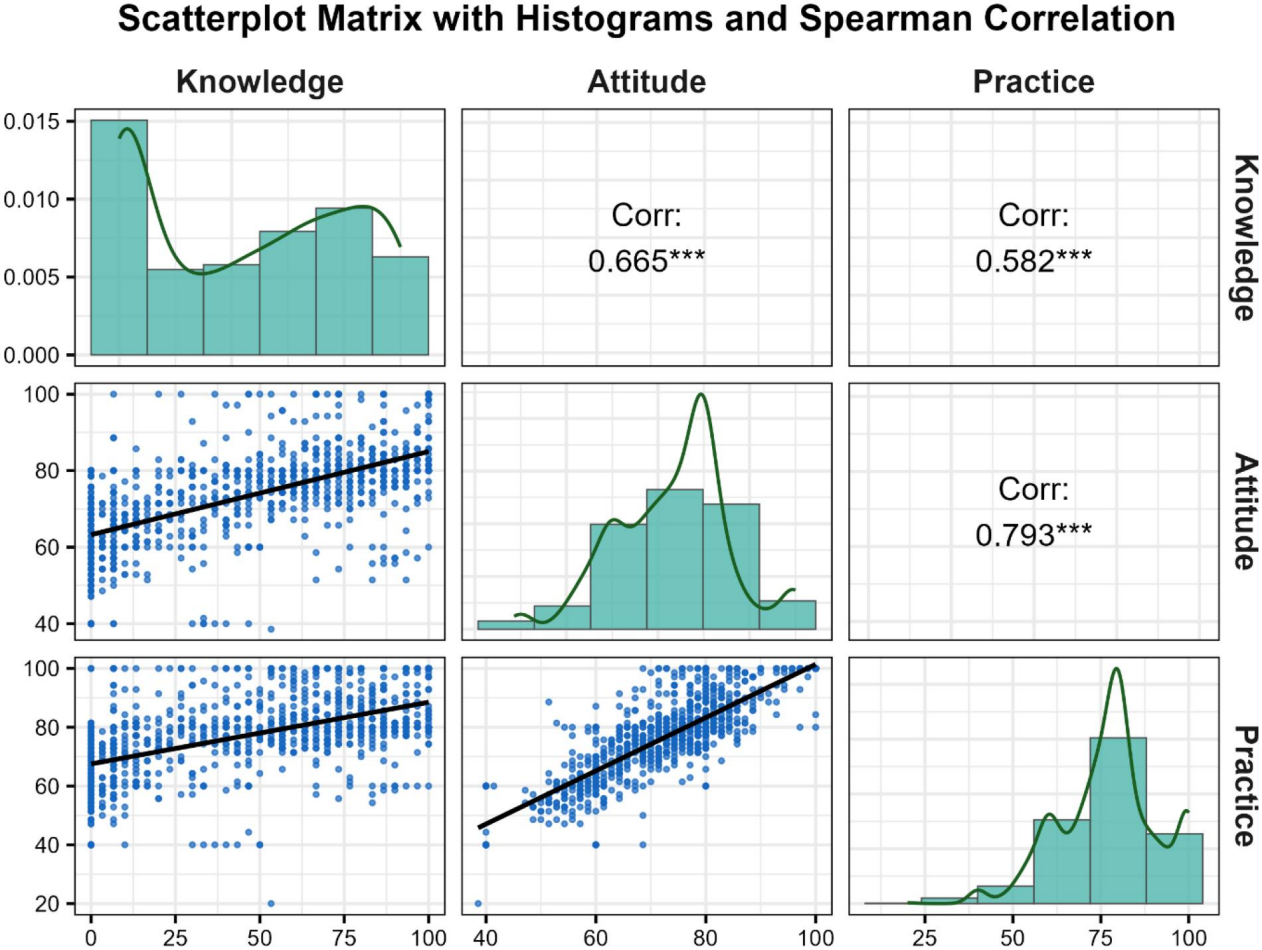
Scatterplot correlation matrix showing the relationships among knowledge, attitude, and practice (KAP) scores regarding Mpox among Bangladeshi respondents (*N* = 1002). Diagonal panels represent the distribution of individual KAP scores, lower panels display scatterplots with fitted trend lines, and upper panels indicate Spearman's correlation coefficients (*ρ*) with corresponding significance levels.

**Table 4:** Spearman's rank correlation among respondents of knowledge, attitude, and practice regarding Mpox.

Association	Correlation (*ρ*)	*p* value
Knowledge and attitudes	0.665	< 0.001
Knowledge and practices	0.582	< 0.001
Attitudes and practices	0.793	< 0.001

The analysis found a significant positive correlation between knowledge and attitude (*ρ* = 0.665, *p* < 0.001) (Table 4). The respondents with greater knowledge were more likely to hold favorable attitudes regarding Mpox prevention and control. Similarly, knowledge was positively linked to practice (*ρ* = 0.582, *p* < 0.001). People with higher knowledge scores also tended to report higher practice scores. The strongest link was observed between attitude and practice (*ρ* = 0.793, *p* < 0.001). Overall, the findings demonstrated that knowledge, attitude, and practice were positively correlated. The strong association between attitude and practice may indicate an important relationship between these domains; however, the cross‐sectional design does not allow the direction or causality of these relationships to be established (Table 4).

## 4 Discussion

Following the COVID‐19 pandemic, the recent outbreak of Mpox represents a potential public health concern globally. Accordingly, this study was designed to assess the KAP of the Bangladeshi population regarding Mpox and to identify key factors associated with these KAP levels. Evidence indicates that adequate knowledge, a positive attitude, and appropriate preventive practices among the mass people during an outbreak can promote a healthier and safer life while reducing stress.

Regarding knowledge, the current study revealed that 37.2% of participants had an adequate level of knowledge regarding Mpox. The observed lower knowledge levels among participants were associated with differences in educational attainment. This finding may reflect differences in access to or exposure to accurate health information regarding Mpox, although such mechanisms were not directly assessed in this study. This finding aligns with similar patterns of awareness reported in a study conducted in Bahir Dar city, Northwest Ethiopia [30], targeting general residents and Bangladesh [31] among university students. However, these levels are lower than those reported in studies conducted in Ghana [37], Uganda [38], China [39], and Jordan [32] among different target groups. Our results are consistent with the study conducted in Pakistan that the higher educational group had significantly better knowledge [40]. Similarly, a preliminary community‐based study in China observed a similar finding pattern in the educational group [39]. The findings of this study suggest that educational attainment is a potential factor in public health preparedness. Consequently, strengthening community‐based health education initiatives may serve as an effective strategy for enhancing awareness and preventing Mpox transmission [29]. Our study revealed that coastal residents had significantly more knowledge regarding Mpox. Several factors may underline this finding. First, Coastal communities are relatively more exposed to zoonotic disease awareness programs due to the greater risk of emerging infectious diseases. Second, Environmental and occupational factors, such as close contact with wildlife and engagement in farming activities, likely increase their risk of exposure, which in turn may enhance their attentiveness to disease prevention measures [41, 42].

Regarding attitude, this study found that 74% of participants exhibited a positive attitude toward Mpox, which is slightly consistent (77%) with the previous study performed in Ghana among university students [37]. Despite participants demonstrating a generally positive attitude toward Mpox prevention, their overall knowledge regarding the disease was found to be limited. This finding underscores the significance of the disease while indicating that participants' understanding of its transmission, clinical manifestations, and preventive measures remains insufficient. The observed discrepancy between knowledge and attitude suggests that a positive attitude alone does not necessarily translate into informed or appropriate behaviors. These findings also underscore the crucial need for comprehensive health education programs to bridge this gap effectively. Comparatively higher levels of attitude were reported in a previous study conducted in Uganda [38]. In constant, 57.3% of the general residents in Ethiopia demonstrated a positive attitude about Mpox [30]. A cross‐sectional study among university students in Bangladesh [31] reported a very low (25.1%) level of attitude, but our study revealed a new finding among mass people in Bangladesh. In the current study, educational attainment was significantly associated with attitudes, with participants in the higher educational group more likely to report positive attitudes. A similar observation has been reported in a previous study carried out in China [39]. In agreement with a multi‐country study of medical students and our current study, a higher level of attitude was observed among urban residents towards Mpox [43].

Concerning practices related to Mpox, 80% of respondents demonstrated appropriate practices. Although the overall knowledge about Mpox was relatively low, a higher proportion of respondents demonstrated appropriate preventive practices. This finding may reflect previous exposure to COVID‐19‐related preventive behaviors, such as hand hygiene, mask use, and social distancing. These previously learned behaviors may partly explain the observed level of Mpox‐related preventive practices, even among those with limited specific knowledge about the disease. Similarly, previous studies have indicated that experiences from past pandemics can enhance preparedness for emerging infectious diseases [37, 43]. A comparatively low level of practice is reported by university students in Bangladesh [31]. Participants from the urban area followed significantly satisfactory practices, and this finding aligns with the previous study [31]. The respondent who had a previous health issue reported satisfactory practice. This association may reflect differences in health care–seeking behavior, health awareness, or social support; however, these potential explanations were not directly assessed in the present study [44, 45]. In the case of the overall KAP score, participants from the higher education group significantly improved their overall KAP scores about Mpox. Participants from the Urban areas showed a higher tendency for the overall correct KAP score.

### 4.1 Strengths and Limitations

This study is one of the first large‐scale, community‐based surveys in Bangladesh to comprehensively assess KAP toward Mpox among the general population. Its wide geographic coverage, inclusion of border, coastal, and central regions, and relatively large sample size enhance the generalizability of findings. However, several limitations should be acknowledged. First, although a multistage sampling approach was used, with sampling across districts, upazilas, and households, the statistical analyses did not explicitly account for the clustered nature of the data. Therefore, standard errors may have been underestimated, and CIs may be narrower than they would be under a cluster‐adjusted analysis. However, the sampling structure and study setting were considered when interpreting the findings, and the observed associations were not interpreted as causal relationships. Second, the use of a 65% cut‐off to categorize KAP scores, although supported by previous literature, may have resulted in some loss of information compared with analyzing KAP scores as continuous variables. Third, the high Cronbach's alpha values observed for the knowledge (0.956), attitude (0.941), and practice (0.959) scales indicate excellent internal consistency but may also reflect some redundancy among questionnaire items. Finally, the cross‐sectional design precludes establishing temporal or causal relationships; therefore, the identified factors should be interpreted as associated factors rather than determinants, and the public‐health implications should be considered accordingly. Despite these limitations, this exploratory study provides useful insights into community KAP regarding Mpox in Bangladesh and may help inform the development of future awareness and preparedness initiatives.

### 4.2 Recommendations

Given the suboptimal knowledge yet positive attitudes and practices observed, community‐based health education and awareness campaigns are recommended, particularly in border areas with low knowledge levels. Targeted interventions should prioritize groups with limited education and day laborers, who demonstrated poorer outcomes. Integrating Mpox awareness into existing public health programs and leveraging lessons from COVID‐19 preparedness may enhance prevention strategies. Strengthening cross‐border and global collaboration is also important for coordinated surveillance and control of emerging zoonotic threats.

## 5 Conclusion

This study provides important community‐based evidence from a relatively large sample on public KAP regarding Mpox in Bangladesh. While the majority of respondents reported positive attitudes and preventive practices, knowledge levels were found to be suboptimal. Education emerged as an important associated factor influencing KAP outcomes, highlighting the critical role of awareness‐building interventions. Notably, populations in border regions and those engaged in low‐income occupations, such as day laborers, were more vulnerable to poor KAP levels. The strong correlations between attitudes and practices emphasize that shaping positive perceptions is central to translating knowledge into effective preventive behaviors. These findings may have important implications for public health preparedness in Bangladesh, where porous borders and ecological overlap with potential reservoirs increase the risk of Mpox introduction. To strengthen national readiness, community‐based awareness campaigns, integration of Mpox education into existing public health programs, and focused interventions targeting high‐risk groups are recommended. Regional and cross‐border collaboration will also be important to enhance surveillance, response, and control strategies for emerging zoonotic threats.

## Author Contributions

**Hemayet Hossain:** conceptualization, data curation, formal analysis, software, investigation, methodology, resources, writing – original draft, writing – review and editing. **Md. Hasan Ali:** formal analysis, investigation, writing – original draft. **Md. Abu Boccor Siddique:** writing – original draft. **Sumaya Shargin Khan:** data curation, investigation. **Milon Mia:** data curation, investigation. **Suchona Akter:** data curation, investigation. **Afroza Sultana Nitu:** data curation, investigation. **Mohammad Nahian Rahman:** data curation, investigation. **Mostafizor Rahman:** data curation, investigation. **Md. Abdur Nur Sakib:** data curation, investigation, writing – original draft. **Md Mosharof Hosen:** data curation, investigation. **Jakia Sultana Koly:** data curation, investigation. **Tanvir Ahmad:** data curation, investigation. **Sadia Islam Ria:** data curation, investigation. **Tanvir Ahmed:** data curation, investigation. **Khadiza Akter Brishty:** data curation, investigation. **Md. Nafij Mashrur:** investigation, methodology, software. **Shihab Ahmed:** formal analysis, software, investigation, methodology. **Md. Shahidur Rahman Chowdhury:** methodology, resources, software, writing – review and editing. **Md. Mahfujur Rahman:** conceptualization, data curation, formal analysis, software, investigation, methodology, resources, project administration, validation, visualization, supervision, writing – original draft, writing – review and editing. All authors have read and approved the final version of the manuscript. Md. Mahfujur Rahman had full access to all of the data in this study and takes complete responsibility for the integrity of the data and the accuracy of the data analysis.

## Funding

The authors have nothing to report.

## Conflicts of Interest

The authors declare no conflicts of interest.

## Transparency Statement

The lead author, Md. Mahfujur Rahman, affirms that this manuscript is an honest, accurate, and transparent account of the study being reported; that no important aspects of the study have been omitted; and that any discrepancies from the study as planned (and, if relevant, registered) have been explained.

## Supporting information


**Checklist S1:** STROBE Statement checklist of items used in this study.

**Questionnaire S1:** Survey Project: Public Awareness, Perceptions, and Preventive Practices Regarding Mpox in Bangladesh.

**Table S1:** Distribution of participants' knowledge regarding Mpox (N = 1,002).

**Table S2:** Distribution of participants' Attitude regarding Mpox (N = 1,002).

**Table S3:** Distribution of participants' practice regarding Mpox (N = 1,002).

**Table S4:** Univariable and multivariable analyses show the association of demographic factors with the knowledge level.

**Table S5:** Univariable and multivariable analyses show the association of demographic factors with the attitude level.

**Table S6:** Univariable and multivariable analyses show the association of demographic factors with the practice level.

## Data Availability

The authors confirm that all data underlying the findings are fully available without restriction. All relevant data are within the paper and its Supporting Information files.
