# Supplementary material for: Public Knowledge, Attitudes, and Preventive Practices Regarding Mpox in Bangladesh: Insights From a Community‐Based Cross‐Sectional Survey
Source: Health Sci Rep. 2026 Oct 16;9(10):e73401. doi: 10.1002/hsr2.73401 (PMC13673291; doi:10.1002/hsr2.73401)
Supplement: Supplementary file 1 [file HSR2-9-e73401-s001.docx]

| **Table S2.** Distribution of participants’ Attitude regarding Mpox (N = 1,002) | |
| --- | --- |
| **Characteristic** | **N = 1,002** |
| **Mpox poses a serious health risk.** |  |
| Strongly Agree | 209 (20.9%) |
| Agree | 403 (40.2%) |
| Neutral | 325 (32.4%) |
| Disagree | 59 (5.9%) |
| Strongly Disagree | 6 (0.6%) |
| **Following proper public health measures can control Mpox effectively.** |  |
| Strongly Agree | 203 (20.3%) |
| Agree | 551 (55.0%) |
| Neutral | 195 (19.5%) |
| Disagree | 48 (4.8%) |
| Strongly Disagree | 5 (0.5%) |
| **Knowing the symptoms of Mpox is important.** |  |
| Strongly Agree | 264 (26.3%) |
| Agree | 486 (48.5%) |
| Neutral | 198 (19.8%) |
| Disagree | 52 (5.2%) |
| Strongly Disagree | 2 (0.2%) |
| **Public awareness campaigns are important to prevent Mpox.** |  |
| Strongly Agree | 241 (24.1%) |
| Agree | 533 (53.2%) |
| Neutral | 194 (19.4%) |
| Disagree | 32 (3.2%) |
| Strongly Disagree | 2 (0.2%) |
| **I am concerned about Mpox spreading in my community.** |  |
| Strongly Agree | 102 (10.2%) |
| Agree | 392 (39.1%) |
| Neutral | 356 (35.5%) |
| Disagree | 130 (13.0%) |
| Strongly Disagree | 22 (2.2%) |
| **Smallpox vaccination may provide protection against Mpox.** |  |
| Strongly Agree | 75 (7.5%) |
| Agree | 289 (28.8%) |
| Neutral | 455 (45.4%) |
| Disagree | 163 (16.3%) |
| Strongly Disagree | 20 (2.0%) |
| **Avoiding contact with wild animals or infected individuals is an effective way to prevent Mpox.** |  |
| Strongly Agree | 97 (9.7%) |
| Agree | 484 (48.3%) |
| Neutral | 319 (31.8%) |
| Disagree | 94 (9.4%) |
| Strongly Disagree | 8 (0.8%) |
| **The government’s efforts to prevent Mpox are insufficient.** |  |
| Strongly Agree | 144 (14.4%) |
| Agree | 446 (44.5%) |
| Neutral | 325 (32.4%) |
| Disagree | 77 (7.7%) |
| Strongly Disagree | 10 (1.0%) |
| **People living near borders are at higher risk of Mpox spread.** |  |
| Strongly Agree | 94 (9.4%) |
| Agree | 452 (45.1%) |
| Neutral | 367 (36.6%) |
| Disagree | 87 (8.7%) |
| Strongly Disagree | 2 (0.2%) |
| **Cross-border collaboration is crucial for effective Mpox control.** |  |
| Strongly Agree | 88 (8.8%) |
| Agree | 418 (41.7%) |
| Neutral | 423 (42.2%) |
| Disagree | 69 (6.9%) |
| Strongly Disagree | 4 (0.4%) |
| **Residents of border areas should be provided with more information on Mpox prevention.** |  |
| Strongly Agree | 133 (13.3%) |
| Agree | 464 (46.3%) |
| Neutral | 314 (31.3%) |
| Disagree | 89 (8.9%) |
| Strongly Disagree | 2 (0.2%) |
| **Migration and travel across borders impact the risk of Mpox outbreaks.** |  |
| Strongly Agree | 95 (9.5%) |
| Agree | 460 (45.9%) |
| Neutral | 376 (37.5%) |
| Disagree | 70 (7.0%) |
| Strongly Disagree | 1 (0.1%) |
| **Border control and movement restrictions are effective in preventing Mpox spread.** |  |
| Strongly Agree | 98 (9.8%) |
| Agree | 464 (46.3%) |
| Neutral | 361 (36.0%) |
| Disagree | 78 (7.8%) |
| Strongly Disagree | 1 (0.1%) |
| **Global collaboration plays a key role in controlling Mpox spread.** |  |
| Strongly Agree | 234 (23.4%) |
| Agree | 467 (46.6%) |
| Neutral | 251 (25.0%) |
| Disagree | 49 (4.9%) |
| Strongly Disagree | 1 (0.1%) |
