# Supplementary material for: Public Knowledge, Attitudes, and Preventive Practices Regarding Mpox in Bangladesh: Insights From a Community‐Based Cross‐Sectional Survey
Source: Health Sci Rep. 2026 Oct 16;9(10):e73401. doi: 10.1002/hsr2.73401 (PMC13673291; doi:10.1002/hsr2.73401)
Supplement: Supplementary file 2 [file HSR2-9-e73401-s002.docx]

| **Table S4.** Univariable and multivariable analyses show the association of demographic factors with the knowledge level | | | | | | | | |
| --- | --- | --- | --- | --- | --- | --- | --- | --- |
|  | **Knowledge level** | | **Univariate LR** | | | **Multivariate LR** | | |
| **Variables** | **Good** N = 373 | **Poor** N = 629 | **COR** | **95% CI** | ***p*-value** | **AOR** | **95% CI** | ***p*-value** |
| **Participant Gender** |  |  |  |  |  |  |  |  |
| Male | 204 (54.7%) | 346 (55%) | Ref. | — |  | — | — |  |
| Female | 169 (45.3%) | 283 (45%) | 1.01 | 0.78, 1.31 | >0.9 | 1.31 | 0.90, 1.91 | 0.2 |
| **Education level** |  |  |  |  |  |  |  |  |
| No formal education | 18 (4.8%) | 190 (30.2%) | Ref. | — |  | — | — |  |
| Primary education | 6 (1.6%) | 96 (15.3%) | 0.66 | 0.23, 1.63 | 0.4 | 0.53 | 0.18, 1.36 | 0.2 |
| Secondary/Higher secondary | 69 (18.5%) | 200 (31.8%) | 3.64 | 2.13, 6.51 | <0.001 | 2.36 | 1.23, 4.69 | 0.012 |
| BSc Hons or Higher degree | 280 (75.1%) | 143 (22.7%) | 20.7 | 12.6, 36.0 | <0.001 | 11.8 | 5.61, 25.8 | <0.001 |
| **Occupation** |  |  |  |  |  |  |  |  |
| Business person | 62 (16.6%) | 94 (14.9%) | Ref. | — |  | — | — |  |
| Day laborer | 22 (5.9%) | 179 (28.5%) | 0.19 | 0.11, 0.32 | <0.001 | 0.88 | 0.44, 1.77 | 0.7 |
| Government Employee | 37 (9.9%) | 33 (5.2%) | 1.70 | 0.96, 3.01 | 0.067 | 1.00 | 0.51, 1.97 | >0.9 |
| Healthcare Provider | 66 (17.7%) | 46 (7.3%) | 2.18 | 1.33, 3.58 | 0.002 | 1.26 | 0.69, 2.32 | 0.5 |
| Housewife | 28 (7.5%) | 151 (24%) | 0.28 | 0.17, 0.47 | <0.001 | 0.70 | 0.31, 1.57 | 0.4 |
| Student | 158 (42.4%) | 126 (20.1%) | 1.90 | 1.28, 2.84 | 0.002 | 1.06 | 0.56, 2.04 | 0.9 |
| **Income Level/month (BDT TK)** |  |  |  |  |  |  |  |  |
| 10000-30000 | 94 (25.2%) | 193 (30.7%) | Ref. | — |  | — | — |  |
| No income | 109 (29.2%) | 224 (35.6%) | 1.00 | 0.71, 1.40 | >0.9 | 0.95 | 0.53, 1.71 | 0.9 |
| Below 10000 | 88 (23.6%) | 151 (24%) | 1.20 | 0.83, 1.72 | 0.3 | 0.89 | 0.49, 1.61 | 0.7 |
| 30000-50000 | 66 (17.7%) | 50 (7.9%) | 2.71 | 1.75, 4.24 | <0.001 | 1.35 | 0.79, 2.29 | 0.3 |
| Over 50000 | 16 (4.3%) | 11 (1.8%) | 2.99 | 1.35, 6.86 | 0.008 | 2.42 | 0.92, 6.65 | 0.077 |
| **Household Size (Number of family members)** |  |  |  |  |  |  |  |  |
| 4-5 | 241 (64.6%) | 348 (55.3%) | Ref. | — |  | — | — |  |
| below 4 | 76 (20.4%) | 87 (13.8%) | 1.26 | 0.89, 1.79 | 0.2 | 1.15 | 0.74, 1.80 | 0.5 |
| 5+ | 56 (15%) | 194 (30.9%) | 0.42 | 0.29, 0.58 | <0.001 | 0.51 | 0.34, 0.77 | 0.002 |
| **Age (Years)** |  |  |  |  |  |  |  |  |
| <20 | 17 (4.6%) | 30 (4.8%) | Ref. | — |  | — | — |  |
| 20-30 | 263 (70.5%) | 259 (41.1%) | 1.79 | 0.98, 3.39 | 0.065 | 0.67 | 0.31, 1.49 | 0.3 |
| 31-40 | 60 (16.1%) | 151 (24%) | 0.70 | 0.36, 1.39 | 0.3 | 0.70 | 0.28, 1.76 | 0.4 |
| 40+ | 33 (8.8%) | 189 (30.1%) | 0.31 | 0.15, 0.63 | <0.001 | 0.44 | 0.17, 1.13 | 0.086 |
| **Participant’s settlement type** |  |  |  |  |  |  |  |  |
| Rural | 131 (35.1%) | 410 (65.2%) | Ref. | — |  | — | — |  |
| Semi-urban | 99 (26.5%) | 91 (14.5%) | 3.40 | 2.41, 4.82 | <0.001 | 1.27 | 0.81, 1.99 | 0.3 |
| Urban | 143 (38.4%) | 128 (20.3%) | 3.50 | 2.57, 4.77 | <0.001 | 1.24 | 0.82, 1.88 | 0.3 |
| **Location Upazila** |  |  |  |  |  |  |  |  |
| Bordered | 74 (19.8%) | 218 (34.6%) | Ref. | — |  | — | — |  |
| Non-bordered | 196 (52.6%) | 264 (42%) | 2.19 | 1.59, 3.03 | <0.001 | 1.13 | 0.75, 1.70 | 0.6 |
| Central | 44 (11.8%) | 32 (5.1%) | 4.05 | 2.40, 6.90 | <0.001 | 1.85 | 0.98, 3.53 | 0.059 |
| Costal | 59 (15.8%) | 115 (18.3%) | 1.51 | 1.00, 2.28 | 0.048 | 2.24 | 1.31, 3.85 | 0.003 |
| **Previous Health Issues (Related to Skin rash, fever, muscle pain)** |  |  |  |  |  |  |  |  |
| No | 218 (58.4%) | 335 (53.3%) | Ref. | — |  | — | — |  |
| Yes | 155 (41.6%) | 294 (46.7%) | 0.81 | 0.62, 1.05 | 0.11 | 1.14 | 0.81, 1.60 | 0.5 |
| **Infected by any type of pox before (or similar signs of Pox)** |  |  |  |  |  |  |  |  |
| No | 117 (31.4%) | 172 (27.3%) | Ref. | — |  | — | — |  |
| Yes | 256 (68.6%) | 457 (72.7%) | 0.82 | 0.62, 1.09 | 0.2 | 1.26 | 0.88, 1.81 | 0.2 |
| AOR, adjusted odds ratio; CI, confidence interval; COR, crude odds ratio. Ref.: Reference Category  P < 0.05 was considered statistically significant. LR: Logistic Regression | | | | | | | | |
