# Supplementary material for: Public Knowledge, Attitudes, and Preventive Practices Regarding Mpox in Bangladesh: Insights From a Community‐Based Cross‐Sectional Survey
Source: Health Sci Rep. 2026 Oct 16;9(10):e73401. doi: 10.1002/hsr2.73401 (PMC13673291; doi:10.1002/hsr2.73401)
Supplement: Supplementary file 3 [file HSR2-9-e73401-s003.docx]

**S1 Questionnaire:** Survey Project: Public Awareness, Perceptions, and Preventive Practices Regarding Mpox in Bangladesh

**Name: ……………………………………………Location: …………………………………….**

**Mobile No. …………………………………………….**

**Section A: Sociodemographic Information**

1. Gender
   ☐ Male
   ☐ Female
2. Age
   ☐ <20
   ☐ 20–30
   ☐ 31–40
   ☐ 40+
3. Education level
   ☐ No formal education
   ☐ Primary education
   ☐ Secondary/Higher secondary
   ☐ BSc Hons or Higher degree
4. Occupation
   ☐ Student
   ☐ Housewife
   ☐ Day laborer
   ☐ Business person
   ☐ Healthcare provider
   ☐ Government employee
5. Income level/month
   ☐ No income
   ☐ Below 10,000 BDT
   ☐ 10,000–30,000 BDT
   ☐ 30,000–50,000 BDT
   ☐ Over 50,000 BDT
6. Household size
   ☐ Below 4
   ☐ 4–5
   ☐ 5+
7. Settlement type
   ☐ Rural
   ☐ Semi-urban
   ☐ Urban
8. Location type
   ☐ Bordered
   ☐ Non-bordered
   ☐ Coastal
   ☐ Central
9. Have you ever experienced health issues like skin rash, fever, muscle pain?
   ☐ Yes
   ☐ No
10. Have you ever been infected by any type of pox (or had similar symptoms)?
    ☐ Yes
    ☐ No

**Section B: Knowledge about Mpox**

(Responses: **Yes / Maybe / No**)

1. Have you ever heard about Mpox (Monkeypox)?
2. Do you know Mpox is a viral disease?
3. Are you aware Mpox outbreaks have occurred globally, including Bangladesh?
4. Do you know which animals can carry Mpox?
5. Do you know Mpox can be transmitted from animals to humans?
6. Do you know Mpox can spread person-to-person by direct contact?
7. Do you know early detection and reporting can help control outbreaks?
8. Do you know Mpox causes fever, rash, headache, muscle pain?
9. Do you know Mpox can cause serious illness in people with weak immunity?
10. Do you know there is no specific treatment, but symptoms can be managed?
11. Do you know smallpox vaccination may provide some protection?
12. Do you know isolating infected people helps prevent spread?
13. Do you know the health guidelines for preventing Mpox?
14. Would you visit a doctor if you suspected Mpox?
15. Are you aware of potential economic impacts of Mpox in your community?

**Section C: Attitudes toward Mpox**

(Responses: **Strongly Agree / Agree / Neutral / Disagree / Strongly Disagree**)

1. Mpox poses a serious health risk.
2. Following proper public health measures can control Mpox.
3. Knowing symptoms of Mpox is important.
4. Public awareness campaigns are essential for prevention.
5. I am concerned about Mpox spreading in my community.
6. Smallpox vaccination may protect against Mpox.
7. Avoiding contact with wild animals or infected people prevents Mpox.
8. Government’s prevention efforts are insufficient.
9. People living near borders are at higher risk.
10. Cross-border collaboration is crucial for control.
11. Border residents should get more prevention information.
12. Migration and travel increase Mpox risk.
13. Border control and restrictions help prevent spread.
14. Global collaboration is key for Mpox control.

**Section D: Preventive Practices regarding Mpox**

(Responses: **Strongly Agree / Agree / Neutral / Disagree / Strongly Disagree**)

1. Hands should be washed after contact with animals or infected people.
2. PPE should be worn when handling animals or infected individuals.
3. Surfaces contaminated by infected individuals should be disinfected.
4. Medical advice should be sought if Mpox symptoms appear.
5. Contact with suspected carriers should be avoided.
6. Health guidelines should be strictly followed.
7. Participation in community health programs is important.
8. Suspected cases should be reported to authorities.
9. Quarantine should be followed if exposed to Mpox.
10. Avoid visiting Mpox-affected regions.
11. Avoid consuming food from contaminated/affected areas.
12. At-risk individuals should receive vaccination.
13. Travel advisories should be followed.
14. Information should be obtained from trusted sources.
