# Supplementary material for: Public Knowledge, Attitudes, and Preventive Practices Regarding Mpox in Bangladesh: Insights From a Community‐Based Cross‐Sectional Survey
Source: Health Sci Rep. 2026 Oct 16;9(10):e73401. doi: 10.1002/hsr2.73401 (PMC13673291; doi:10.1002/hsr2.73401)
Supplement: Supplementary file 4 [file HSR2-9-e73401-s004.docx]

| **Table S5.** Univariable and multivariable analyses show the association of demographic factors with the attitude level. | | | | | | | | |
| --- | --- | --- | --- | --- | --- | --- | --- | --- |
|  | **Attitude level** | | **Univariate LR** | | | **Multivariate LR** | | |
| **Variables** | **Negative** N = 261 | **Positive** N = 741 | **COR** | **95% CI** | ***p*-value** | **AOR** | **95% CI** | ***p*-value** |
| **Participant Gender** |  |  |  |  |  |  |  |  |
| Male | 153 (58.6%) | 397 (53.6%) | Ref. | — |  | — | — |  |
| Female | 108 (41.4%) | 344 (46.4%) | 1.23 | 0.92, 1.64 | 0.2 | 1.41 | 0.84, 2.40 | 0.2 |
| **Education level** |  |  |  |  |  |  |  |  |
| No formal education | 120 (46%) | 88 (11.9%) | Ref. | — |  | — | — |  |
| Primary education | 45 (17.2%) | 57 (7.7%) | 1.73 | 1.07, 2.80 | 0.025 | 1.14 | 0.66, 1.98 | 0.6 |
| Secondary/Higher secondary | 73 (28%) | 196 (26.4%) | 3.66 | 2.50, 5.40 | <0.001 | 1.58 | 0.94, 2.64 | 0.083 |
| BSc Hons or Higher degree | 23 (8.8%) | 400 (54%) | 23.7 | 14.6, 40.0 | <0.001 | 5.68 | 2.63, 12.5 | <0.001 |
| **Occupation** |  |  |  |  |  |  |  |  |
| Business person | 43 (16.5%) | 113 (15.2%) | Ref. | — |  | — | — |  |
| Day laborer | 104 (39.8%) | 97 (13%) | 0.35 | 0.23, 0.55 | <0.001 | 0.86 | 0.46, 1.61 | 0.6 |
| Government Employee | 8 (3.1%) | 62 (8.4%) | 2.95 | 1.37, 7.12 | 0.009 | 1.24 | 0.50, 3.32 | 0.7 |
| Healthcare Provider | 5 (1.9%) | 107 (14.5%) | 8.14 | 3.39, 24.2 | <0.001 | 2.98 | 1.06, 9.83 | 0.051 |
| Housewife | 72 (27.6%) | 107 (14.5%) | 0.57 | 0.35, 0.89 | 0.015 | 0.98 | 0.41, 2.30 | >0.9 |
| Student | 29 (11.1%) | 255 (34.4%) | 3.35 | 2.00, 5.68 | <0.001 | 1.90 | 0.80, 4.49 | 0.14 |
| **Income Level/month (BDT TK)** |  |  |  |  |  |  |  |  |
| 10000-30000 | 89 (34.1%) | 198 (26.7%) | Ref. | — |  | — | — |  |
| No income | 100 (38.3%) | 233 (31.5%) | 1.05 | 0.74, 1.48 | 0.8 | 0.64 | 0.33, 1.23 | 0.2 |
| Below 10000 | 60 (23%) | 179 (24.1%) | 1.34 | 0.91, 1.98 | 0.14 | 0.97 | 0.54, 1.73 | >0.9 |
| 30000-50000 | 10 (3.8%) | 106 (14.3%) | 4.76 | 2.48, 10.1 | <0.001 | 1.50 | 0.67, 3.56 | 0.3 |
| Over 50000 | 2 (0.8%) | 25 (3.4%) | 5.62 | 1.63, 35.4 | 0.021 | 3.66 | 0.81, 26.6 | 0.13 |
| **Household Size (Number of family members)** |  |  |  |  |  |  |  |  |
| 4-5 | 141 (54%) | 448 (60.5%) | Ref. | — |  | — | — |  |
| below 4 | 23 (8.8%) | 140 (18.9%) | 1.92 | 1.21, 3.16 | 0.008 | 1.67 | 0.94, 3.07 | 0.088 |
| 5+ | 97 (37.2%) | 153 (20.6%) | 0.50 | 0.36, 0.68 | <0.001 | 0.94 | 0.62, 1.45 | 0.8 |
| **Age (Years)** |  |  |  |  |  |  |  |  |
| <20 | 9 (3.5%) | 38 (5.1%) | Ref. | — |  | — | — |  |
| 20-30 | 72 (27.6%) | 450 (60.7%) | 1.48 | 0.65, 3.07 | 0.3 | 1.03 | 0.37, 2.72 | >0.9 |
| 31-40 | 69 (26.4%) | 142 (19.2%) | 0.49 | 0.21, 1.02 | 0.071 | 1.01 | 0.33, 2.97 | >0.9 |
| 40+ | 111 (42.5%) | 111 (15%) | 0.24 | 0.10, 0.49 | <0.001 | 0.64 | 0.21, 1.88 | 0.4 |
| **Participant’s settlement type** |  |  |  |  |  |  |  |  |
| Rural | 221 (84.7%) | 320 (43.2%) | Ref. | — |  | — | — |  |
| Semi-urban | 16 (6.1%) | 174 (23.5%) | 7.51 | 4.51, 13.4 | <0.001 | 2.19 | 1.19, 4.20 | 0.014 |
| Urban | 24 (9.2%) | 247 (33.3%) | 7.11 | 4.61, 11.4 | <0.001 | 2.28 | 1.34, 3.97 | 0.003 |
| **Location Upazila** |  |  |  |  |  |  |  |  |
| Bordered | 115 (44.1%) | 177 (23.9%) | Ref. | — |  | — | — |  |
| Non-bordered | 60 (23%) | 400 (54%) | 4.33 | 3.04, 6.23 | <0.001 | 3.07 | 1.99, 4.79 | <0.001 |
| Central | 7 (2.7%) | 69 (9.3%) | 6.40 | 3.03, 15.8 | <0.001 | 2.30 | 0.94, 6.34 | 0.084 |
| Costal | 79 (30.2%) | 95 (12.8%) | 0.78 | 0.53, 1.14 | 0.2 | 0.93 | 0.57, 1.51 | 0.8 |
| **Previous Health Issues (Related to Skin rash, fever, muscle pain)** |  |  |  |  |  |  |  |  |
| No | 139 (53.3%) | 414 (55.9%) | Ref. | — |  | — | — |  |
| Yes | 122 (46.7%) | 327 (44.1%) | 0.90 | 0.68, 1.20 | 0.5 | 2.02 | 1.36, 3.02 | <0.001 |
| **Infected by any type of pox before (or similar signs of Pox)** |  |  |  |  |  |  |  |  |
| No | 59 (22.6%) | 230 (31%) | Ref. | — |  | — | — |  |
| Yes | 202 (77.4%) | 511 (69%) | 0.65 | 0.46, 0.90 | 0.010 | 0.71 | 0.45, 1.10 | 0.13 |
| AOR, adjusted odds ratio; CI, confidence interval; COR, crude odds ratio. Ref.: Reference Category  P < 0.05 was considered statistically significant. LR: Logistic Regression | | | | | | | | |
