# Supplementary material for: Public Knowledge, Attitudes, and Preventive Practices Regarding Mpox in Bangladesh: Insights From a Community‐Based Cross‐Sectional Survey
Source: Health Sci Rep. 2026 Oct 16;9(10):e73401. doi: 10.1002/hsr2.73401 (PMC13673291; doi:10.1002/hsr2.73401)
Supplement: Supplementary file 6 [file HSR2-9-e73401-s006.docx]

| **Table S1.** Distribution of participants’ knowledge regarding Mpox (N = 1,002) | |
| --- | --- |
| **Characteristic** | **N = 1,002** |
| **Have you ever heard about Mpox (Monkeypox)?** |  |
| Yes | 544 (54.3%) |
| Maybe | 36 (3.6%) |
| No | 422 (42.1%) |
| **Do you know that Mpox is a viral disease?** |  |
| Yes | 374 (37.3%) |
| Maybe | 72 (7.2%) |
| No | 556 (55.5%) |
| **Are you aware that Mpox outbreaks have occurred globally, including in Bangladesh?** |  |
| Yes | 418 (41.7%) |
| Maybe | 103 (10.3%) |
| No | 481 (48.0%) |
| **Do you know which animals can carry Mpox?** |  |
| Yes | 327 (32.6%) |
| Maybe | 70 (7.0%) |
| No | 605 (60.4%) |
| **Do you know that Mpox can be transmitted from animals to humans?** |  |
| Yes | 406 (40.5%) |
| Maybe | 101 (10.1%) |
| No | 495 (49.4%) |
| **Do you know Mpox can spread from person to person by direct contact?** |  |
| Yes | 411 (41.0%) |
| Maybe | 138 (13.8%) |
| No | 453 (45.2%) |
| **Do you know that early detection and reporting of Mpox can help control outbreaks?** |  |
| Yes | 415 (41.4%) |
| Maybe | 174 (17.4%) |
| No | 413 (41.2%) |
| **Do you know that Mpox can cause fever, rash, headache, and muscle pain?** |  |
| Yes | 390 (38.9%) |
| Maybe | 118 (11.8%) |
| No | 494 (49.3%) |
| **Do you know Mpox can cause serious illness in people with weak immunity?** |  |
| Yes | 341 (34.0%) |
| Maybe | 178 (17.8%) |
| No | 483 (48.2%) |
| **Do you know there is no specific treatment for Mpox, but symptoms can be managed?** |  |
| Yes | 318 (31.7%) |
| Maybe | 195 (19.5%) |
| No | 489 (48.8%) |
| **Do you know that smallpox vaccination may provide some protection against Mpox?** |  |
| Yes | 225 (22.5%) |
| Maybe | 212 (21.2%) |
| No | 565 (56.4%) |
| **Do you know isolating infected people can help prevent Mpox spread?** |  |
| Yes | 420 (41.9%) |
| Maybe | 193 (19.3%) |
| No | 389 (38.8%) |
| **Do you know the health guidelines for preventing Mpox?** |  |
| Yes | 227 (22.7%) |
| Maybe | 112 (11.2%) |
| No | 663 (66.2%) |
| **Would you visit a doctor if you thought you had Mpox?** |  |
| Yes | 587 (58.6%) |
| Maybe | 134 (13.4%) |
| No | 281 (28.0%) |
| **Are you aware of the potential economic impact of an MPOX outbreak in your community?** |  |
| Yes | 404 (40.3%) |
| Maybe | 179 (17.9%) |
| No | 419 (41.8%) |
