# Supplementary material for: Public Knowledge, Attitudes, and Preventive Practices Regarding Mpox in Bangladesh: Insights From a Community‐Based Cross‐Sectional Survey
Source: Health Sci Rep. 2026 Oct 16;9(10):e73401. doi: 10.1002/hsr2.73401 (PMC13673291; doi:10.1002/hsr2.73401)
Supplement: Supplementary file 7 [file HSR2-9-e73401-s007.docx]

| **Table S6.** Univariable and multivariable analyses show the association of demographic factors with the practice level | | | | | | | | |
| --- | --- | --- | --- | --- | --- | --- | --- | --- |
|  | **Practice level** | | **Univariate LR** | | | **Multivariate LR** | | |
| **Variables** | Satisfactory N = 802 | Unsatisfactory N = 200 | **COR** | **95% CI** | ***p*-value** | **AOR** | **95% CI** | ***p*-value** |
| **Participant Gender** |  |  |  |  |  |  |  |  |
| Male | 422 (52.6%) | 128 (64%) | Ref. | — |  | — | — |  |
| Female | 380 (47.4%) | 72 (36%) | 1.60 | 1.17, 2.21 | 0.004 | 2.18 | 1.17, 4.16 | 0.016 |
| **Education level** |  |  |  |  |  |  |  |  |
| No formal education | 98 (12.2%) | 110 (55%) | Ref. | — |  | — | — |  |
| Primary education | 76 (9.5%) | 26 (13%) | 3.28 | 1.97, 5.61 | <0.001 | 2.42 | 1.34, 4.46 | 0.004 |
| Secondary/Higher secondary | 218 (27.2%) | 51 (25.5%) | 4.80 | 3.20, 7.27 | <0.001 | 1.95 | 1.12, 3.41 | 0.019 |
| BSc Hons or Higher degree | 410 (51.1%) | 13 (6.5%) | 35.4 | 19.8, 68.4 | <0.001 | 6.49 | 2.62, 16.8 | <0.001 |
| **Occupation** |  |  |  |  |  |  |  |  |
| Business person | 125 (15.6%) | 31 (15.5%) | Ref. | — |  | — | — |  |
| Day laborer | 106 (13.2%) | 95 (47.5%) | 0.28 | 0.17, 0.44 | <0.001 | 0.42 | 0.20, 0.84 | 0.015 |
| Government Employee | 64 (8.0%) | 6 (3.0%) | 2.65 | 1.12, 7.32 | 0.039 | 0.96 | 0.34, 3.02 | >0.9 |
| Healthcare Provider | 109 (13.6%) | 3 (1.5%) | 9.01 | 3.11, 38.3 | <0.001 | 2.39 | 0.68, 11.3 | 0.2 |
| Housewife | 128 (16%) | 51 (25.5%) | 0.62 | 0.37, 1.03 | 0.068 | 0.46 | 0.17, 1.24 | 0.13 |
| Student | 270 (33.6%) | 14 (7.0%) | 4.78 | 2.50, 9.57 | <0.001 | 2.40 | 0.82, 7.18 | 0.11 |
| **Income Level/month (BDT TK)** |  |  |  |  |  |  |  |  |
| 10000-30000 | 207 (25.8%) | 80 (40%) | Ref. | — |  | — | — |  |
| No income | 267 (33.3%) | 66 (33%) | 1.56 | 1.08, 2.27 | 0.019 | 0.86 | 0.41, 1.83 | 0.7 |
| Below 10000 | 192 (23.9%) | 47 (23.5%) | 1.58 | 1.05, 2.39 | 0.029 | 1.33 | 0.70, 2.54 | 0.4 |
| 30000-50000 | 110 (13.7%) | 6 (3.0%) | 7.09 | 3.24, 18.7 | <0.001 | 1.64 | 0.64, 4.80 | 0.3 |
| Over 50000 | 26 (3.3%) | 1 (0.5%) | 10.0 | 2.08, 181 | 0.025 | 4.49 | 0.69, 89.6 | 0.2 |
| **Household Size (Number of family members)** |  |  |  |  |  |  |  |  |
| 4-5 | 482 (60.1%) | 107 (53.5%) | Ref. | — |  | — | — |  |
| below 4 | 142 (17.7%) | 21 (10.5%) | 1.50 | 0.92, 2.54 | 0.11 | 1.24 | 0.65, 2.42 | 0.5 |
| 5+ | 178 (22.2%) | 72 (36%) | 0.55 | 0.39, 0.78 | <0.001 | 1.25 | 0.78, 2.03 | 0.4 |
| **Age (Years)** |  |  |  |  |  |  |  |  |
| <20 | 42 (5.2%) | 5 (2.5%) | Ref. | — |  | — | — |  |
| 20-30 | 475 (59.2%) | 47 (23.5%) | 1.20 | 0.40, 2.93 | 0.7 | 1.50 | 0.39, 5.23 | 0.5 |
| 31-40 | 150 (18.7%) | 61 (30.5%) | 0.29 | 0.10, 0.71 | 0.013 | 1.35 | 0.32, 5.19 | 0.7 |
| 40+ | 135 (16.9%) | 87 (43.5%) | 0.18 | 0.06, 0.44 | <0.001 | 1.37 | 0.33, 5.23 | 0.7 |
| **Participant’s settlement type** |  |  |  |  |  |  |  |  |
| Rural | 363 (45.3%) | 178 (89%) | Ref. | — |  | — | — |  |
| Semi-urban | 181 (22.6%) | 9 (4.5%) | 9.86 | 5.21, 21.2 | <0.001 | 2.17 | 1.02, 5.09 | 0.056 |
| Urban | 258 (32.1%) | 13 (6.5%) | 9.73 | 5.63, 18.3 | <0.001 | 2.29 | 1.20, 4.65 | 0.016 |
| **Location Upazila** |  |  |  |  |  |  |  |  |
| Bordered | 207 (25.8%) | 85 (42.5%) | Ref. | — |  | — | — |  |
| Non-bordered | 426 (53.1%) | 34 (17%) | 5.14 | 3.37, 8.01 | <0.001 | 3.84 | 2.30, 6.51 | <0.001 |
| Central | 71 (8.9%) | 5 (2.5%) | 5.83 | 2.50, 17.1 | <0.001 | 1.47 | 0.52, 4.84 | 0.5 |
| Costal | 98 (12.2%) | 76 (38%) | 0.53 | 0.36, 0.78 | 0.001 | 0.48 | 0.28, 0.81 | 0.006 |
| **Previous Health Issues (Related to Skin rash, fever, muscle pain)** |  |  |  |  |  |  |  |  |
| No | 448 (55.9%) | 105 (52.5%) | Ref. | — |  | — | — |  |
| Yes | 354 (44.1%) | 95 (47.5%) | 0.87 | 0.64, 1.19 | 0.4 | 2.01 | 1.28, 3.19 | 0.003 |
| **Infected by any type of pox before (or similar signs of Pox)** |  |  |  |  |  |  |  |  |
| No | 238 (29.7%) | 51 (25.5%) | Ref. | — |  | — | — |  |
| Yes | 564 (70.3%) | 149 (74.5%) | 0.81 | 0.57, 1.15 | 0.2 | 0.94 | 0.56, 1.55 | 0.8 |
| Abbreviations: AOR, adjusted odds ratio; CI, confidence interval; COR, crude odds ratio. Ref.: Reference Category,  P < 0.05 was considered statistically significant. LR: Logistic Regression | | | | | | | | |
