# Supplementary material for: Public Knowledge, Attitudes, and Preventive Practices Regarding Mpox in Bangladesh: Insights From a Community‐Based Cross‐Sectional Survey
Source: Health Sci Rep. 2026 Oct 16;9(10):e73401. doi: 10.1002/hsr2.73401 (PMC13673291; doi:10.1002/hsr2.73401)
Supplement: Supplementary file 8 [file HSR2-9-e73401-s008.docx]

| **Table S3.** Distribution of participants’ practice regarding Mpox (N = 1,002) | |
| --- | --- |
| **Characteristic** | **N = 1,002** |
| **Hands should be washed after contact with carrier animals or infected individuals.** |  |
| Strongly Agree | 335 (33.4%) |
| Agree | 451 (45.0%) |
| Neutral | 177 (17.7%) |
| Disagree | 37 (3.7%) |
| Strongly Disagree | 2 (0.2%) |
| **Personal protective equipment (PPE) should be worn when handling animals or infected individuals.** |  |
| Strongly Agree | 229 (22.9%) |
| Agree | 389 (38.8%) |
| Neutral | 309 (30.8%) |
| Disagree | 68 (6.8%) |
| Strongly Disagree | 7 (0.7%) |
| **Surfaces such as floors, beds, and yards potentially contaminated by animals or infected individuals should be disinfected.** |  |
| Strongly Agree | 241 (24.1%) |
| Agree | 455 (45.4%) |
| Neutral | 250 (25.0%) |
| Disagree | 55 (5.5%) |
| Strongly Disagree | 1 (0.1%) |
| **Medical advice should be sought if symptoms resembling Mpox are experienced.** |  |
| Strongly Agree | 241 (24.1%) |
| Agree | 495 (49.4%) |
| Neutral | 216 (21.6%) |
| Disagree | 49 (4.9%) |
| Strongly Disagree | 1 (0.1%) |
| **Contact with animals or infected individuals known to carry Mpox should be avoided.** |  |
| Strongly Agree | 218 (21.8%) |
| Agree | 486 (48.5%) |
| Neutral | 227 (22.7%) |
| Disagree | 68 (6.8%) |
| Strongly Disagree | 3 (0.3%) |
| **Health authority guidelines for Mpox prevention should be strictly followed.** |  |
| Strongly Agree | 263 (26.2%) |
| Agree | 480 (47.9%) |
| Neutral | 207 (20.7%) |
| Disagree | 51 (5.1%) |
| Strongly Disagree | 1 (0.1%) |
| **People should participate in community health programs related to Mpox.** |  |
| Strongly Agree | 176 (17.6%) |
| Agree | 482 (48.1%) |
| Neutral | 292 (29.1%) |
| Disagree | 50 (5.0%) |
| Strongly Disagree | 2 (0.2%) |
| **Suspected Mpox cases should be reported to health authorities.** |  |
| Strongly Agree | 196 (19.6%) |
| Agree | 512 (51.1%) |
| Neutral | 231 (23.1%) |
| Disagree | 62 (6.2%) |
| Strongly Disagree | 1 (0.1%) |
| **Quarantine measures should be followed if exposed to Mpox.** |  |
| Strongly Agree | 221 (22.1%) |
| Agree | 429 (42.8%) |
| Neutral | 276 (27.5%) |
| Disagree | 73 (7.3%) |
| Strongly Disagree | 3 (0.3%) |
| **People should refrain from visiting regions where Mpox cases have been reported.** |  |
| Strongly Agree | 204 (20.4%) |
| Agree | 480 (47.9%) |
| Neutral | 245 (24.5%) |
| Disagree | 71 (7.1%) |
| Strongly Disagree | 2 (0.2%) |
| **Avoid consuming food from sources that may be contaminated or from**  **Mpox-affected areas.** |  |
| Strongly Agree | 174 (17.4%) |
| Agree | 430 (42.9%) |
| Neutral | 287 (28.6%) |
| Disagree | 106 (10.6%) |
| Strongly Disagree | 5 (0.5%) |
| **At-risk individuals should be encouraged to receive Mpox vaccination.** |  |
| Strongly Agree | 173 (17.3%) |
| Agree | 443 (44.2%) |
| Neutral | 297 (29.6%) |
| Disagree | 85 (8.5%) |
| Strongly Disagree | 4 (0.4%) |
| **Will you follow all recommended Mpox travel advisories?** |  |
| Strongly Agree | 199 (19.9%) |
| Agree | 481 (48.0%) |
| Neutral | 268 (26.7%) |
| Disagree | 51 (5.1%) |
| Strongly Disagree | 3 (0.3%) |
| **Accurate and up-to-date information about Mpox should be obtained from trusted sources.** |  |
| Strongly Agree | 362 (36.1%) |
| Agree | 408 (40.7%) |
| Neutral | 201 (20.1%) |
| Disagree | 29 (2.9%) |
| Strongly Disagree | 2 (0.2%) |
